# Syphilis epidemiology in Norway, 1992-2008: resurgence among men who have sex with men

**DOI:** 10.1186/1471-2334-10-105

**Published:** 2010-04-29

**Authors:** Irena Jakopanec, Andrej M Grjibovski, Øivind Nilsen, Preben Aavitsland

**Affiliations:** 1 Department of Infectious Disease Epidemiology, Norwegian Institute of Public Health, PO Box 4404 Nydalen, N-0403 Oslo, Norway; 2 Institute of Community Medicine, University of Tromsø, Tromsø, Norway; 3 International School of Public Health, Northern State Medical University, Arkhangelsk, Russia

## Abstract

**Background:**

In recent years, the number of syphilis cases has stabilised in many countries of Western Europe, however several countries have reported increases among men who have sex with men (MSM). The aim of this article was to describe the epidemiology of early syphilis in Norway in 1992-2008.

**Methods:**

Cases of early syphilis and congenital syphilis reported to the Norwegian Surveillance System for Communicable Diseases (MSIS) 1992-2008 were described by route of transmission, gender, age, birthplace, stage of disease, HIV co-infection, source partner and place of infection.

**Results:**

The incidence of reported syphilis ranged from 0.05 (1992) to 1.50 (2002) per 100 000 person-years. Of 562 cases reported to MSIS during the study period, 62% were men infected by another man. The proportion of those, infected homosexually increased from 0 (1992-1994) to 77% (2008). Most of them were Norwegians (83%). The proportion of HIV co-infection among homosexually infected increased over time and reached 39% in 2008. The majority reported being infected by a casual partner (73%) and in the municipality of Oslo (72%). Of 152 heterosexually infected men 64% were Norwegians; 51% were infected by casual contacts and 20% by commercial sex workers; 73% were infected abroad. Among 56 women, 57% were Norwegians, 57% were infected by a steady partner and 40% were infected abroad. Almost half (46%) were diagnosed in the early latent stage. Four cases had congenital syphilis, two of whom were adopted from abroad.

**Conclusions:**

Syphilis is rare in Norway, but MSM represent almost two thirds of cases. The increase of HIV co-infected cases among MSM may enhance transmission of both infections. We recommend sexually active MSM to be tested for syphilis 2-4 times a year. Due to its variable clinical course, syphilis might be difficult to recognise at an early stage among women in a low-prevalence population. We estimate current practice of prenatal screening in Norway as sufficient.

## Background

Following increases in the early 2000s in Western European countries, the number of reported syphilis cases has recently stabilised; however many countries reported a high proportion of homosexually acquired syphilis (France, Denmark, Ireland, Germany, UK, Sweden, Netherlands) [1]. Increases among men who have sex with men (MSM) have been reported worldwide [2] and a significant proportion of them have been found to be co-infected with HIV [3,4].

Infectious stages of syphilis are primary, denoted by a painless ulcer in about one third of patients, and secondary, with diverse symptoms. Patients in the latent stage are seemingly unaffected, but about 25% may experience relapses to secondary syphilis during the early latent stage (i.e. less than a year since infection) [4,5]. Vertical transmission may result in congenital syphilis, difficult to recognise in seemingly asymptomatic newborns [6]. Untreated, syphilis may result in death and serious disability [4,5], however effective penicillin-based treatment is inexpensive. Testing and treatment are free of charge in Norway.

Serological tests for syphilis lack sensitivity and though some may be helpful in assessing the stage of infection, they are unreliable [7]. In determining the stage, particularly in asymptomatic patients, clinicians thus rely on previously documented syphilis tests, anamnesis (i.e. most likely time of exposure, data from contact tracing, known previous treatment) and clinical evaluation. Uncertain stage in asymptomatic patients with unknown duration of infection is an important limitation of early syphilis (i.e. primary, secondary and early latent syphilis) surveillance.

Using data from the Norwegian Surveillance System for Communicable Diseases (MSIS), we describe the epidemiology of early syphilis in Norway from 1992-2008.

## Methods

Under the Infectious Disease Control Act, syphilis is a mandatory notifiable disease, therefore all clinicians and laboratories in Norway must notify all cases of newly diagnosed syphilis anonymously to the MSIS, based at the Norwegian Institute of Public Health (NIPH). Contact tracing and notification, mandatory by the same act, is the responsibility of the clinicians. Previously mandatory screening of pregnant women in Norway became optional in 1995; however, virtually all pregnant women are still tested. If a patient has a positive syphilis test, screening for other sexually transmitted infections (STIs) is recommended.

Upon a positive syphilis test, all 22 local laboratories in Norway send their notification to both NIPH and the clinician involved. Both of these notification forms carry the same unique non-identifying number for an individual patient. Having received notification from the laboratory, clinicians fill out the corresponding clinical report on the stage of the disease, patient's demographic data, symptoms, co-existing STIs, risk behaviour (including most likely transmission route and time of exposure) and their source partner, and send it to the NIPH. There, only one co-existing STI can be entered into the MSIS database. If the patient is reported to have an HIV infection, HIV is always entered.

At NIPH, only newly recognised cases with early syphilis, defined as laboratory confirmed, primary, secondary or early latent syphilis (less than a year since infection) are entered into the MSIS database. The reports on a single patient from laboratories and clinicians are merged using the unique non-identifying number. Every reported case is individually evaluated at NIPH and clinicians can be contacted if the stage of syphilis is unclear or if they failed to report on a case, notified by laboratory only.

We used the following key variables from the reports: route of transmission, gender, month and year of birth, birthplace (country and continent), place of residence, reason for being in Norway, indications for testing, duration of symptoms, stage of disease, co-existent STI, relation to the source person, place of infection and the type of clinical practice where the diagnose was made. We also studied differences between HIV co-infected and HIV negative men, infected with syphilis homosexually. The data were analysed in categories as presented in Table 1.

**Table 1:** Selected characteristics of sexually infected cases with early syphilis reported to the Norwegian surveillance system for communicable diseases, absolute numbers, (N = 558), 1992-2008.

Characteristic		Selected categories	Sex			P1*	P2*
					
			Women n = 56 (%)	Men			
						
				Heterosexual transmission n = 152 (%)	Homosexual transmission n = 350 (%)		
Age		Median age in years	26	37	37	/	/
		
		15-24 years	22 (39.3)	8 (5.3)	33 (9.4)	0.222	< 0.001
				
		25-34 years	16 (28.6)	56 (36.8)	103 (29.4)		
				
		35-44 years	13 (23.2)	47 (30.9)	119 (34.0)		
				
		≥ 45 years	5 (8.9)	41 (27.0)	95 (27.1)		

Residence		Oslo municipality	16 (28.6)	55 (36.2)	266 (76.0)	< 0.001	0.304
				
		Other	40 (71.4)	97 (63.8)	84 (24.0)		

Birthplace		Norway	32 (57.1)	97 (63.8)	290 (82.8)	< 0.001	0.773
				
		Europe, other	8 (14.3)	20 (12.7)	28 (8.0)		
				
		Asia	11 (19.6)	24 (15.8)	12 (3.4)		
				
		Africa	3 (5.3)	9 (5.9)	6 (1.7)		
				
		Other	2 (3.6)	2 (1.3)	14 (4.0)		

Reason for being in Norway		Temporary visit	0	2 (1.3)	9 (2.6)	0.546	0.644
				
		First generation immigrant/adopted	5 (8.9)	16 (10.5)	30 (8.6)		
				
		Other, including permanent residents	51 (91.1)	134 (88.1)	311 (88.9)		

Indications for testing		Symptoms	15 (26.8)	121 (79.6)	248 (70.8)	< 0.001	< 0.001**
				
		Contact tracing	17 (30.3)	12 (7.9)	29 (8.3)		
				
		Own request	1 (1.8)	5 (3.3)	25 (7.1)		
				
		Routine testing of an immigrant	6 (10.7)	6 (3.9)	0		
				
		Pregnancy	13 (23.2)	/	/		
				
		No specific reason/other	4 (7.1)	8 (5.3)	48 (13.7)		

Median duration of symptoms in days*** (interquartile range)			23 (19-47)	22 (10-56)	22 (9-44)	/	/

Stage of early syphilis		Primary	18 (32.1)	84 (55.3)	125 (35.7)	< 0.001	< 0.001
				
		Secondary	12 (21.4)	43 (28.3)	149 (42.6)		
				
		Early latent	26 (46.4)	25 (16.4)	76 (21.7)		

Other reported STI****		none	52 (92.8)	140 (92.1)	239 (68.3)	< 0.001	0.959
				
		HIV	1 (1.8)	2 (1.3)	85 (24.3)		
				
		Chlamydia	1 (1.8)	4 (2.6)	16 (4.6)		
				
		gonorrhoea	0	0	3 (0.9)		
				
		herpes	0	2 (1.3)	3 (0.9)		
				
		hepatitis B	1 (1.8)	2 (1.3)	3 (0.9)		
				
		other	1 (1.8)	2 (1.3)	1 (0.3)		

Source partner		Steady partner	32 (57.1)	20 (13.1)	56 (16.0)	< 0.001	< 0.001
				
		Casual partner	11 (19.6)	78 (51.3)	254 (72.6)		
				
		Commercial sex worker	0	31 (20.4)	0		
				
		Other/Unknown	13 (23.2)	23 (15.1)	40 (11.4)		

Place of infection	Abroad	Total abroad	23 (41.1)	111 (73.0)	69 (19.7)		
				
		Europe, other	8 (14.3)	43 (28.3)	57 (16.3)	< 0.001*****	0.001*****
				
		- In Russia	3 (5.4)	20 (13.1)	1 (0.3)		
				
		Asia	9 (16.1)	34 (22.4)	4 (1.1)		
				
		Africa	3 (5.4)	11 (7.2)	2 (0.6)		
				
		South/Mid America	2 (3.6)	18 (11.8)	4 (1.1)		
				
		Other	1 (1.8)	5 (3.3)	2 (0.6)		
			
	Norway	Total Norway	31 (55.4)	33 (21.7)	270 (77.1)		
				
		- In Oslo	7 (12.5)	17 (11.2)	244 (69.7)		
			
	Unknown		2 (3.6)	8 (5.3)	11 (3.1)		

Diagnosed by		General practitioner, private specialist	32 (57.1)	71 (46.7)	85 (24.3)	< 0.001	0.440
				
		Hospital	7 (12.5)	18 (11.8)	55 (15.7)		
				
		Youth/STI clinic	15 (26.8)	59 (38.8)	210 (60.0)		
				
		Other	2 (3.6)	4 (2.6)	0		

*P-values for heterogeneity, using Pearson's chi squared test; P1 comparing homosexually and heterosexually infected men, P2 comparing women and heterosexually infected men

**Women diagnosed during pregnancy were excluded for comparison

*** Data available for 19 women, 109 men, infected heterosexually, and 199 men, infected homosexually

**** Only one co-infection can be recorded into the system: if reported, HIV is always recorded

***** Seven categories were compared, including: Norway, Europe-other, Asia, Africa, South/Mid America, other (abroad), and unknown

The data on all cases of early or congenital syphilis were obtained from MSIS. The size of population used for calculating incidence rate for each year was obtained from Statistics Norway [8] and was about 4.5 million. We used Stata 9.2 software (STATA Corp., TX, USA) for the analysis. Simple linear regression analysis was used to estimate trends over time. Given that the data between subsequent years may be correlated and the variability between the years may not be constant over time, the regression coefficients and their 95% confidence intervals (CI) were calculated using the Newey-West procedure. Bivariate comparisons between groups were performed using Pearson's chi squared test. Due to different mode of transmission, cases of congenital syphilis were described separately.

## Results

From 1992 to 2008, 562 cases of syphilis diagnosed in Norway were reported to MSIS, including four congenital cases.

**Figure 1:**
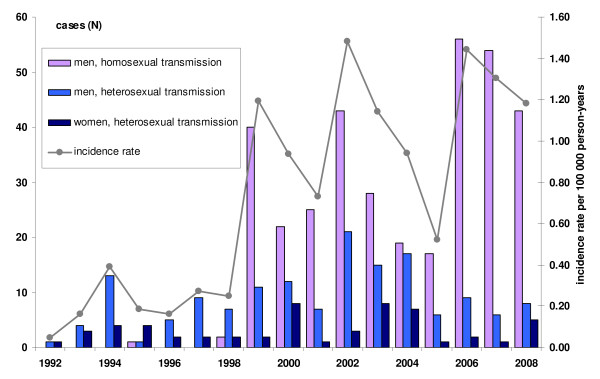
**Number of early syphilis cases by sexual transmission routes in Norway (N = 558), 1992-2008**.

The incidence rate in the study period was 0.7 per 100 000 person-years (95% CI: 0.06-1.0); varying from 0.05 per 100 000 person-years in 1992 to 1.2 per 100 000 person-years in 2008. A peak was observed in 2002 with a rate of 1.5 per 100 000 person-years (Figure 1). On average, there was an increase of 3.8 cases each year (95% CI: 2.8-4.8, p for trend < 0.001). No sexually infected case was younger than 15 years. Of the 502 (89%) men who were infected sexually, 350 (70%) were MSM.

### Men, infected homosexually

**Figure 2:**
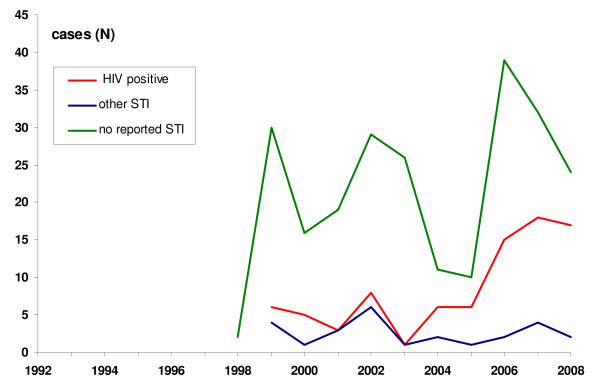
**Men, homosexually infected with early syphilis, according to simultaneously reported HIV and other STI, Norway (N = 350), 1992-2008**. Only one STI co-infection is recorded: if HIV is reported, it is always recorded.

One man, infected homosexually in 1995, and 2 in 1998 were followed by a marked increase of 40 cases in 1999, representing 75% of all reported cases (Figure 1). More than a half (61%) of the total 350 cases were older than 34 years. The majority (76%) resided in Oslo and 83% were born in Norway. Before testing, 71% had symptoms. The largest proportion of cases was diagnosed in a secondary stage (43%). Almost one quarter of these men were also HIV positive (Table 1). The proportion of those co-infected with HIV increased over time (p for trend < 0.001) and reached 39% in 2008 (Figure 2). This increasing trend remained significant even if calculated from 1999, when HIV co-infected were first reported (p for trend = 0.009). Most homosexually infected men were infected by a casual partner (73%) and in Oslo municipality (72%), (Table 1). Among 69 infected abroad, 18 (5% of all cases) were infected in Spain.

Men, homosexually infected with syphilis, who were also HIV positive, were more likely to be residents of Oslo (p = 0.001), being diagnosed with syphilis later in the course of disease (primary vs. secondary or early latent, p = 0.006) and in a hospital (p < 0.001) than HIV negative, but they did not differ by age, birthplace, indications for syphilis testing, source partner and place of infection.

### Men, infected heterosexually

During the study period, 152 men were infected heterosexually. No obvious time trend was observed (p for trend = 0.158). More than half (57%) were older than 34 years. Most (64%) were Norwegian, with a notable proportion reporting a birthplace in Asia (16%). The majority (80%) reported symptoms and their infection was mostly discovered in the primary stage. Two men were HIV positive. Casual contacts and commercial sex workers (CSW) were the source of infection in 71%. Most acquired their infection abroad (73%) (Table 1); predominantly in Russia (13%), Pakistan (10%), Brazil (7%) and Thailand (6%). Most Norwegians were infected abroad, mainly in other countries of Europe (36%), Asia (18%) and South and Mid America (16%). Immigrants were mainly infected during travels back to their continent of birth.

### Women

Among 56 women reported to be infected with syphilis from 1992-2008, 68% were younger than 35 years. Most of the cases were Norwegian (57%) and 55% acquired their infection in Norway. However, out of 31 infected in Norway, 14 reported their male partner had been infected abroad. Contact tracing was the predominant cause for testing in women (30%), followed by symptoms (27%) and pregnancy (23%). Almost half (46%) of all cases were discovered in the early latent stage of disease. STI co-infections were rare (Table 1). Women reported a steady partner as the source of infection in 57%.

### Children with congenital syphilis

The age of the four patients with congenital syphilis at the time of diagnosis ranged from one to five years, one of them was a boy. Two of these children were adopted from abroad. The biological mother of one child was born in Norway, but acquired her infection abroad, while the mother of the other child was infected in Norway and tested positive with her second pregnancy in 2003, when the affected child was five years old.

## Discussion

Syphilis is a rare disease in Norway. However, since 1999, there has been a resurgence among MSM, disproportionately affecting HIV positive men. The epidemic among MSM is mainly concentrated in the capital Oslo. Up to 73% of heterosexual men and 41% of women reported being infected abroad. Compared to men, women seem to be more frequently diagnosed as late as the early latent stage and are diagnosed predominantly by contact tracing. Men infected heterosexually reported being infected by casual partner or a CSW in 71% of cases, as opposed to women, who appear to be mostly infected by their steady partners.

One of the strengths of our study is a probable high coverage of diagnosed patients in MSIS [9,10] due to double notifications from both laboratories and clinicians and a long tradition of reporting among the latter. A large set of variables on each patient is available; allowing for demographic and behavioural insight.

Our study is subject to several limitations. Due to the clinical course of this disease, some infected may stay undiagnosed. Others, discovered by routine testing and possibly asymptomatic, need to be evaluated by a clinician for stage. We cannot completely exclude, however, that some patients, reported as "early latent syphilis" may have been infected longer than a year. This would particularly affect data on women in our study. Contrary to this, every year, about hundred cases are only lab-reported. Clinicians are aware we only include new cases of early syphilis in the database, as we publish this information in a yearly national MSIS report [11]. Contrary to our experience with gonorrhoea with high reporting from both laboratories and clinicians [9], clinicians may opt out from sending in their part of the reporting form for syphilis, if the person has been infected for more than a year or has been known to have a positive test from before. If the stage is unknown or in cases, only reported by the laboratories, internal evaluation at NIPH includes the testing sites (asylum centres), the immigrant status and unknown date of exposure of most of these cases, to determine the likelihood of late syphilis, however it may happen there are some missed early cases among them. Clinicians may also be contacted by NIPH to verify information. Furthermore, behaviour data is subject to response and recall bias. Data in a few variables are frequently missing, including co-infection data. We believe, however, that reporting is reliable on HIV co-infection. Capacity for entering several co-infections at a time should be improved in MSIS. The fact that hardly any cases were reported as homosexually acquired until 1999 cannot be explained by changes in the notification system or improved sexual behaviour reporting as in comparison, 10-42 cases of homosexually acquired gonorrhoea per year were reported in the same time period and in the same surveillance system [12].

Among European countries with various systems of syphilis surveillance, the highest incidence rates for early syphilis in 2007 (per 100 000 person-years) were reported from: the UK (4.4, primary and secondary stages only), Czech Republic (4.0), Sweden (2.6, less than two yeas since infection), Germany (2.5) [1,13]. At NIPH, no total counts of reported syphilis cases are kept, but late latent probably represent a majority (more than 50%). In the period from 2002-2007, late cases represented 50-73% of all reported cases in Czech Republic, 35-51% in Germany and 24-50% in Slovenia [1].

Compared to infected women in our study, heterosexual men were older, more likely to be diagnosed in a primary stage and to report symptoms, and less likely to be diagnosed through contact tracing. Similar findings were reported from a London-based study [14]. Men are more likely than women to report having been infected abroad, which resembles the situation in Sweden, however women in Sweden acquired their infection abroad more frequently and had a higher median age (33 years) than women in Norway [13]. A prevalence of HIV co-infection among heterosexuals, much higher than in our study (from 6-13%), has been reported from London and France [14,15]. Compared to London with 12% of heterosexual men reporting being clients of CSW [14], a higher proportion of men in Norway used commercial sex services, however they did so mostly abroad.

Similar to many other European countries [16], the epidemics among MSM in Scandinavia are mainly concentrated in men above 30 in metropolitan areas [13,17]. A high, but stable proportion of syphilis and HIV co-infections among MSM in several countries has been ascribed to serosorting, practice of oral sex and compromised immune system [14,17,18]. While information on co-infection is not always available, others have also reported a simultaneous increase of HIV and syphilis among MSM from 2000-2005 [19,20]. HIV positive MSM were also less likely to be in the primary stage of syphilis when diagnosed as reported from London Enhanced Syphilis Surveillance programme (2001-2004) [14].

The increase of pharyngeal, frequently asymptomatic STIs among MSM has been associated with the practice of unprotected oral sex, which is perceived as low risk for HIV transmission [12,18,21]. This might explain the differences in syphilis stage at the time of diagnosis between homosexually and heterosexually infected men, as initial painless lesion ("chancre"), appearing in the oropharynx (as well as the rectum) may pass unnoticed among MSM. Differences in syphilis stage between serodiscordant homosexual men may be explained by potentially asymptomatic disease among the HIV positive [19] and a possible overlap of the primary and secondary stage [4]. Genital ulcers, including syphilitic chancre, can facilitate HIV acquisition and transmission [22], which may, together with frequent partner exchange, explain a simultaneous increase of both infections among MSM.

Compared to official population data, foreign-born people are over-represented among syphilis cases in Norway [23]. The reason could be opportunistic testing of immigrants originating from countries with higher syphilis prevalence. Occasional increases of syphilis cases, linked to CSW, refugees/asylum seekers, immigrants from the eastern Europe, especially Russia, or travelling to these regions, were reported by several countries (Czech Republic, Slovenia, Finland, UK) [24-26]. In Norway, only sporadic syphilis cases related to Russia with limited secondary transmission were noted [27]. It needs to be emphasized, however, that sexual contact abroad is a key risk factor for heterosexually-acquired syphilis in Norway.

Whether or not a proportion of reported cases with late syphilis represents a public health problem we should further focus on in Norway, is debatable. As noted, the majority of these cases are immigrants from countries with high syphilis prevalence and were infected in countries of their origin many years ago. On the other hand, mild, unspecific and painless syphilis symptoms make it difficult to diagnose the disease early, especially in women or MSM, who may not notice initial lesions in difficult-to-visualise areas [4]. The rarity of the disease in Norway makes it a diagnostic challenge. It is therefore important to focus diagnostic efforts on specific population groups such as MSM and immigrants and, to find women cases, to conduct thorough contact tracing.

An evaluation of the recommendations for yearly syphilis testing of HIV positive MSM in the Netherlands revealed up to third of infections were asymptomatic and only discovered by screening [19]. Clinical care of HIV positive MSM, such as CD4 T cell count or HIV viral load, can conveniently include syphilis testing [28]. We recommend that the epidemic of syphilis among MSM, concentrated in Oslo, be tackled by enhanced syphilis testing for sexually active MSM, whenever they present at the STI clinic or at a general practitioner, ideally 2-4 times a year [29].

The evidence for cost-effectiveness of prenatal screening policy in a very low-risk population (< 1%) seems to be contradictory [30] and universal screening does not prevent mothers from being infected later in the pregnancy. We estimate current recommendations and practice in Norway as sufficient.

## Conclusions

In summary, syphilis in Norway is mainly transmitted among MSM in Oslo. Increasing co-infection with HIV in this group underlines the need for enhanced screening and prevention programmes. We recommend sexually active MSM to be tested 2-4 times a year. Among heterosexuals, coincidental import from other countries has been observed, but further spread in Norway was limited.

## Competing interests

The authors declare that they have no competing interests.

## Authors' contributions

IJ drafted the manuscript. ØN collected and entered data and contributed with interpretation. IJ, AG and PA contributed to the design of the study, analysis and interpretation. All authors critically reviewed and approved the final version of this paper for publication. PA is the guarantor.

## Pre-publication history

The pre-publication history for this paper can be accessed here:

http://www.biomedcentral.com/1471-2334/10/105/prepub
